# Circulating atrial natriuretic peptide genetic association study identifies a novel gene cluster associated with reduced NT-proANP, increased stroke and higher diastolic blood pressure

**DOI:** 10.1186/2050-6511-16-S1-A37

**Published:** 2015-09-02

**Authors:** Naveen L Pereira, Nirubol Tosakulwong, Christopher G Scott, Gregory D Jenkins, Naresh Prodduturi, Yubo Chai, Timothy M Olson, Richard J Rodeheffer, Margaret M Redfield, Richard M Weinshilboum, John C Burnett

**Affiliations:** 1 Cardiorenal Research Laboratory, Division of Cardiovascular Diseases, and Department of Health Sciences Research, Mayo Clinic, Rochester, MN 55905, USA

## Introduction

The goal of this study was to identify genetic determinants of plasma N-terminal proatrial natriuretic peptide (NT-proANP) in the general community by performing a large-scale genetic association study and to assess its functional significance in in vitro cell studies and on disease susceptibility.

## Methods and results

Genotyping was performed across 16,000 genes in 893 randomly selected individuals, with replication in 891 subjects from the community. Plasma NT-proANP1–98 concentrations were determined using a radioimmunoassay. Thirty-three genome-wide significant single-nucleotide polymorphisms were identified in the MTHFR-CLCN6-NPPANPPB locus and were all replicated. To assess the significance, in vitro functional genomic studies and clinical outcomes for carriers of a single-nucleotide polymorphism rs5063 (V32M) located in NPPA that represented the most significant variation in this genetic locus were assessed. The rs5063 variant allozyme in transfected HEK293 cells was decreased to 55±8% of wild-type protein (p=0.01) as assessed by quantitative western blots. Carriers of rs5063 had lower NT-proANP levels (1427 versus 2291 pmol/L; P<0.001) and higher diastolic blood pressures (75 versus 73 mm Hg; p=0.009) and were at an increased risk of stroke when compared with wild-type subjects independent of age, sex, diabetes mellitus, hypertension, atrial fibrillation, and cholesterol levels (hazard ratio, 1.6; p=0.004).

## Conclusion

This is the first large-scale genetic association study of circulating NT-proANP levels performed with replication and functional assessment that identified genetic variants in the MTHFR-CLCN6-NPPA-NPPB cluster to be significantly associated with NT-proANP levels. The clinical significance of this variation is related to lower NT-proANP levels, higher blood pressures, and an increased risk of stroke in the general community.

